# The Fixed Patella Test: A Reliable and Accurate Test for Detecting Quadriceps Tendon Rupture

**DOI:** 10.7759/cureus.93717

**Published:** 2025-10-02

**Authors:** Arifur Rahman, Ahmed Ashour, Ahmed Hamed, Anastasios P Nikolaides, Tarek Boutefnouchet

**Affiliations:** 1 Trauma and Orthopaedics, Walsall Healthcare NHS Trust, Walsall, GBR; 2 Trauma and Orthopaedics, University Hospitals Birmingham NHS Foundation Trust, Birmingham, GBR

**Keywords:** diagnostic, fixed patella test, patella, quadriceps tendon, tendon rupture

## Abstract

Purpose: Delayed diagnosis of quadriceps tendon rupture can compromise outcomes, particularly when standard clinical tests are limited by pain, swelling, or compensatory muscle activation. The fixed patella test is a novel, non-invasive clinical manoeuvre designed to assess tendon continuity. This service evaluation aimed to assess its diagnostic performance in routine clinical practice.

Methods: A prospective service evaluation was conducted at a major UK trauma centre across multiple sites. Patients presenting with suspected extensor mechanism injury underwent routine clinical evaluation, including the fixed patella test. During the test, patients performed an isometric quadriceps contraction while the examiner assessed mediolateral patellar mobility. A mobile patella indicated a ruptured tendon. All participants underwent confirmatory imaging with ultrasound and/or MRI. Surgical findings were used where available.

Results: Thirty patients (31 knees) were included. The fixed patella test demonstrated a sensitivity of 100%, a specificity of 50%, a positive predictive value of 93.1%, a negative predictive value of 100%, and an overall accuracy of 93.5%. All patients with a positive test were confirmed to have a complete or high-grade partial rupture on imaging or at surgery.

Conclusion: The fixed patella test is a simple, highly sensitive, and well-tolerated clinical tool for detecting quadriceps tendon rupture. It requires no additional equipment and may expedite diagnostic confirmation and surgical intervention. Ongoing data collection will help further validate its use in larger cohorts.

## Introduction

The quadriceps tendon, comprising the rectus femoris, vastus lateralis, vastus intermedius, and vastus medialis muscles, plays a critical role in the knee extensor mechanism and is essential for functional activities such as walking, standing, and climbing stairs [1]. Rupture of the quadriceps tendon is relatively uncommon, with an incidence of approximately 1.37 per 100,000 individuals annually, as reported in a UK-based population study [2,3]. These injuries typically occur in men over the age of 40 and are often associated with systemic comorbidities such as chronic kidney disease, diabetes mellitus, and rheumatoid arthritis [4,5].

Although large-scale epidemiological data remain limited, the true incidence may be underreported and is expected to rise with an ageing yet increasingly active population [6]. Prompt diagnosis and timely surgical intervention, ideally within 72 hours, are essential to re-establish extensor continuity, prevent muscle atrophy, and optimise functional recovery [7]. Imaging modalities like ultrasound and MRI provide definitive confirmation, but may not always be immediately available, leading to diagnostic delays.

There is therefore a clinical need for examination techniques that can be rapidly applied at the bedside. The fixed patella test is a novel clinical manoeuvre proposed to address this gap. This multi-centre service evaluation aimed to assess the diagnostic performance of the fixed patella test by determining its sensitivity, specificity, positive predictive value (PPV), and negative predictive value (NPV) in patients with suspected quadriceps tendon rupture. We hypothesised that the fixed patella test would prove to be a reliable and accurate adjunct to routine clinical assessment.

## Materials and methods

This prospective service evaluation was conducted across multiple hospitals within University Hospitals Birmingham NHS Foundation Trust. The project was registered with the trust’s clinical governance department. The fixed patella test was performed as part of routine assessment of patients presenting with suspected quadriceps tendon rupture. Data were anonymised prior to analysis and managed in accordance with GDPR and institutional policy.

Eligible patients were identified through daily review of trauma admissions and assessed either in the emergency department or on inpatient wards. The fixed patella test was performed by trauma and orthopaedic registrars or the study lead. All clinicians received standardised training, which included both visual demonstrations and video-based instruction. Patients who did not undergo the test prior to operative repair were excluded.

Patients were eligible if they were aged 18 years or older and had sustained an acute knee injury with a mechanism consistent with eccentric quadriceps loading. Additional requirements included an inability to perform a straight leg raise, the presence of a palpable defect superior to the patella, and a high clinical suspicion of quadriceps tendon injury. Only patients with the capacity to provide informed consent were included.

Patients were excluded if they had a history of previous quadriceps tendon surgery, underlying neuromuscular disorders, or chronic ruptures older than six weeks. Other exclusion factors included established muscle inhibition, concurrent injuries such as patellar tendon rupture or tibial tubercle avulsion, and ipsilateral femoral, patellar, or tibial fractures. Patients were also excluded if they were unable to tolerate confirmatory imaging with MRI or ultrasound, or if they withdrew consent at any stage.

Index test

The fixed patella test involves the following steps: (i) The patient is asked to perform a sustained isometric quadriceps contraction; (ii) The examiner attempts to move the patella mediolaterally; (iii) The test is repeated on the contralateral patella for comparison. A patella that remains immobile ("fixed") during contraction suggests an intact tendon; a mobile patella indicates a rupture.

Reference standard

All patients underwent confirmatory imaging using ultrasound and/or MRI. Imaging was interpreted by musculoskeletal radiologists who were blinded to the fixed patella test results.

Statistical analysis

Diagnostic accuracy of the fixed patella test was assessed using a 2×2 contingency table. Sensitivity, specificity, PPV, NPV, and overall accuracy were calculated.

## Results

Study population

Between August 2023 and August 2024, 39 consecutive patients with suspected quadriceps tendon rupture were screened. After exclusions (two for delayed presentation/neuromuscular disease; seven due to test non-adherence), 30 patients (31 knees) met eligibility criteria. The cohort comprised predominantly male patients (90%, n=27/30), with a mean age of 57.6±11.4 years (range: 43-85). Ruptures were left-sided in 18 cases (58.1%) and right-sided in 13 (41.9%), as seen in Table 1.

**Table 1:** Cohort demographics and clinical features

Variable	Value
Age, mean ± SD (years)	57.6 ± 11.4
Sex, male:female	27:3
Side of rupture, n (%)	
- Left	18 (58.1%)
- Right	13 (41.9%)
Confirmatory imaging, n	
- MRI	15
- Ultrasound	13
- Both	3

Diagnostic performance of the fixed patella test

Against the reference standard (surgical/imaging confirmation), the fixed patella test demonstrated: true positives: 27; true negatives: 2; false positives (FP): 2; false negatives: 0. Key performance metrics are detailed in Table 2.

**Table 2:** Diagnostic accuracy of the fixed patella test

Metric	Value (%, 95% CI)
Sensitivity	100.0 (87.5–100.0)
Specificity	50.0 (15.0–85.0)
Accuracy	93.5 (79.3–98.2)
Positive predictive value (PPV)	93.1 (78.0–98.1)
Negative predictive value (NPV)	100.0 (34.2–100.0)

The test achieved perfect sensitivity (100%) and high NPV (100%), reliably ruling out rupture when negative. Specificity was moderate (50.0%, 95% CI: 15.0-85.0), reflecting two FP (patients with intact tendons but mobile patellae).

## Discussion

Jolles et al. described a clinical “needle test” for diagnosing quadriceps tendon rupture, in which a needle is inserted into the quadriceps tendon and its movement is observed as the patient flexes the knee. If the needle remains immobile during knee flexion, a ruptured quadriceps tendon is likely [4]. However, there are obvious challenges with this technique, including patient factors like needle phobia and pain on insertion, as well as risks of infection, bleeding, or even needle breakage within the tendon [4]. Furthermore, McGrory suggested aspirating the haematoma from a ruptured quadriceps tendon and injecting local anesthetic to allow extension and assessment of the integrity of the quadriceps [8]. In comparison, the fixed patella test is non-invasive, painless, and requires no additional equipment. This new test avoids the complications associated with needles while providing a quick clinical assessment for quadriceps tendon integrity.

Previous literature suggests that commonly used clinical examinations have limitations. For instance, Siwek and Rao noted that many partial ruptures go undiagnosed when clinicians rely solely on finding a palpable gap-such gaps can be masked by factors like muscle tone or subcutaneous fat [9]. Moreover, Perfitt et al. reported that a palpable defect may not always be evident, particularly in cases of partial or chronic quadriceps tendon ruptures. Swelling and patient habitus can obscure the classic suprapatellar gap, leading to missed or delayed diagnoses [10]. Furthermore, the most commonly used clinical test, the straight leg raise, can be mimicked by recruiting the iliotibial band by slight internal rotation at the hip [11]. These findings in the literature underscore the need for improved clinical tests to detect quadriceps tendon injuries.

Importantly, traditional examination techniques like palpating for a gap or testing straight leg raise should not be abandoned. Instead, the fixed patella test should be used alongside these standard exams to improve diagnostic accuracy. Because of its ease of use, it can easily be incorporated into the clinical evaluation prior to ordering confirmatory imaging. Its use in conjunction with other physical exam findings may raise the index of suspicion for quadriceps tendon rupture even when other signs are equivocal. In a condition where studies have documented misdiagnosis rates of up to 50%, adding an additional clinical test before proceeding to ultrasound (USS) or MRI could help reduce the likelihood of missed injuries [12].

Our findings indicate that the fixed patella test is highly sensitive for detecting quadriceps tendon ruptures. Despite its high sensitivity, the test showed a specificity of only about 50%, indicating a significant rate of false positives. This underscores the importance of confirming the diagnosis with imaging modalities, such as USS or MRI, when the clinical picture is unclear [13]. While imaging is time-consuming and expensive, its role remains critical, especially if physical exam results are inconclusive or potentially misleading. Clinicians should maintain a low threshold for imaging in ambiguous cases, even if the fixed patella test (or other clinical tests) is positive, to ensure accurate diagnosis and timely management.

There are some limitations to our study. Notably, the fixed patella test was performed on only 30 patients. However, considering that this patient cohort was collected over one year across a major trauma centre, the modest sample size highlights how infrequently this injury is encountered in the UK. This rarity may explain the lack of existing specialized techniques for assessing quadriceps tendon ruptures. Our study is ongoing, and we are continuing to collect data on the fixed patella test. We aim to report results from a larger sample in the future and to evaluate the test’s ability to detect partial versus complete quadriceps tendon ruptures. This additional data will help determine the full clinical utility of the fixed patella test and whether it can reliably distinguish the extent of tendon injury.

## Conclusions

The fixed patella test can accurately detect quadriceps tendon rupture. The test was proven to be easy, reliable and very well tolerated by patients, even acutely, since no knee movement is induced. The present study recommends the use of this simple clinical test to help expedite the surgical management of ruptured quadriceps tendons when indicated.
